# The mortality, modes of infection, diagnostic tests, and treatments of Marburg virus disease: A systematic review

**DOI:** 10.1002/hsr2.1545

**Published:** 2023-08-31

**Authors:** Deekshitha Alla, Sai Sri Hari Paruchuri, Angad Tiwari, Sai Santhosha Mrudula Alla, Rakesh Thulaseedharan Pillai, Sandeep Kumar Reddy Bandakadi, Anju Pradeep, Dhruv Jayeshkumar Shah, Mert Sabıroğlu, Sachi Chavda, Patrick Biziyaremye

**Affiliations:** ^1^ Department of Medicine Andhra Medical College Visakhapatnam Andhra Pradesh India; ^2^ Dr. Pinnamaneni Siddhartha Institute of Medical Sciences and Research Foundation China Avutapalle Andhra Pradesh India; ^3^ Maharani Laxmi Bai Medical College Jhansi Uttar Pradesh India; ^4^ SUT Academy of Medical Sciences Kerala India; ^5^ Department of Medicine Osmania Medical College Hyderabad Telangana India; ^6^ Kasturba Medical College Manipal India; ^7^ Emilio Aguinaldo College Manila Philippines; ^8^ Koç University School of Medicine Istanbul Turkey; ^9^ GMERS Medical College Ahmedabad Gujarat India; ^10^ Department of General Medicine University of Rwanda Kigali Rwanda

**Keywords:** general medicine, global health, infectious diseases

## Abstract

**Background and Aims:**

Marburg virus (MARV) has regularly affected people since 1967 causing multiple outbreaks. There are presently no authorized therapies for the fatal Marburg virus disease (MVD), which poses an imminent risk to global public health. The MVD has so far claimed the lives of numerous people, with an increased number of cases being seen throughout the African continent. Hence, a review was carried out to analyze the geographical distribution of MVD, mortality, routes of transmission, and diagnostic and treatment modalities.

**Methods:**

PubMed, Scopus, Web of Science, Google Scholar, and ProMED servers were used to conduct a systematic search in compliance with the PRISMA guidelines. The results were tabulated and analyzed.

**Results:**

A total of 11 studies (7 case reports and 4 case series) were included in the final analysis, and 21 cases of MVD were analyzed. The most frequent symptoms were fever (66.67%), vomiting (57.14%), headache (52.38%), diarrhea (52.38%), and pain (47.62%). The most commonly used diagnostic test was RT‐PCR (42.11%). Contact transmission (50%) and zoonotic transmission (37.5%) were the most prevalent routes of transmission. Antibiotics (61.5%) were the first line of treatment. The most common complications were hemorrhage (60%) and coagulopathies (33.3%). The mortality rate was 57.1%.

**Conclusion:**

To avoid disastrous consequences, it is essential to reiterate the necessity of early diagnosis and treatment of MVD.

## Key Points

- The study provides a detailed analysis of clinical presentation, modes of transmission, complications, diagnostic methods, and treatment modalities of Marburg virus disease (MVD).
- The results of the systematic review supports the high mortality rate of MVD.
- Various countries affected in different outbreaks are enlisted in the study.

## 1 BACKGROUND

On March 21, 2023, the Ministry of Health of the United Republic of Tanzania declared an outbreak of Marburg virus disease (MVD) in the country. As of March 22, a total of eight cases, including five deaths, had been reported in the country.^1^ Another outbreak of MVD occurred in Guinea in February 2023 accounting for 9 laboratory‐confirmed and 20 probable cases. All the probable cases and seven laboratory‐confirmed cases were reported dead.^2^

MVD is a rare but severe hemorrhagic fever that affects both humans and nonhuman primates. MVD is caused by the MARV^3^ (negative‐sense RNA virus, family *Filoviridae*,^4^ closely related to the Ebola virus).^5^

The virus was first identified in 1967 in Marburg and Frankfurt, Germany, and Belgrade, Yugoslavia.^6^, ^7^ Since then, sporadic outbreaks have been reported in Africa.^3^ The most significant outbreaks were in the Democratic Republic of the Congo (DRC) and Angola, with a case fatality rate of 83% and 90%, respectively.^3^

The incubation period is between 2 and 21 days, following which Individuals typically develop sudden illness symptoms such as fever, headache, chills, myalgia, diarrhea, and vomiting, followed by the failure of multiple organs.^8^, ^9^ The virus is transmitted primarily from bats to humans. Human‐to‐human transmission is possible via blood or body fluids, objects contaminated by a sick person, and semen (may be found for up to 407 days^10^) from a man who recovered from MVD. The case‐fatality rate for MVD is between 23% and 90%.^3^

This systematic review aims to provide a comprehensive overview of the various diagnostic and treatment modalities and analyze recovery patterns and routes of transmission of the MARV. By synthesizing the available evidence, this review aims to identify gaps in knowledge and highlight areas for future research.

## 2 METHODS

The Systematic review was carried out according to the PRISMA (Preferred Reporting Items for Systematic Reviews and Meta‐Analyses) guidelines.^11^ The review was registered on PROSPERO (ID: CRD42023420685).

### 2.1 Data sources and search strategy

A comprehensive search was conducted in PubMed, Scopus, and Web of Science databases without publication period restriction. The keywords used for the search were “Marburg virus disease,” “Marburg virus,” “Marburg hemorrhagic fever,” and “African Marburg virus.” A manual search was performed in Google Scholar, the ProMED Mail database (http://www.promedmail.org/), and reference lists.

### 2.2 Eligibility criteria

The studies were included or excluded as per the defined inclusion and exclusion criteria. Case reports or case series of patients affected by the MARV with demographic information and outcome, were included in the study. The cases included had at least one of these components: diagnostic test, route of transmission, clinical presentation, or treatment. We considered the following exclusion criteria:

1. Nonoriginal studies, including conference abstracts, review articles, protocols, and editorials.
2. Articles in a language other than English.
3. Cases without demographic data and outcome.Unavailability of full texts.

### 2.3 Study selection

Revman software was used to organize the search results and remove duplicates. Eight authors independently screened 1785 nonduplicated records and the conflicts were resolved after a discussion with DA, SSM, and PB.

### 2.4 Data extraction

Required data were extracted by eight authors of the research team as follows: first author name, place of study, age gender, diagnostic method, route of transmission, clinical features, complications, treatment, duration of treatment, and outcome. The results of included articles are discussed in Table 1. The first author investigated the extracted data and settled any disagreements among the other authors.

**Table 1:** Table representing patient characteristics, diagnostic methods, route of transmission, treatment, and outcome.

		Paper	Place of study	Route of transmission	Clinical features	Complications/organ system dysfunction	Treatment and duration (days)	Outcome
Geisbert et al.^12^	Kenya	15/M	Histochemistry	N/A	Fever, malaise, haematochezia, vomiting, anorexia, headache	Leucocytosis, prolonged PT, prolonged PTT, DIC, adrenal gland necrosis, hemorrhages in the liver, hemorrhages in the lungs, necrosis, and hemorrhages in the spleen, embolic foci in the kidneys, embolic foci in the heart, microabscesses in the pancreas	Antibiotics, steroids, heparin, fresh plasma, blood transfusions (11)	Death
Smith et al.^13^	Kenya	56/M	Electron microscopy of renal tissue, indirect fluorescent antibody test	Exposure to bats	Fever, headache, myalgia, malaise, diarrhea, vomiting, hematemesis, jaundice	Gastrointestinal hemorrhage, bleeding from the nose and mouth, hepatic necrosis, hemorrhagic diathesis, fulminating hepatitis with hemorrhage	Antibiotics, rehydration (4)	Death
Timen et al.^14^	Netherlands	41/F	RT‐PCR	Exposure to bats	Fever, chills, rash, conjunctivitis, diarrhea	Liver failure, renal failure, hemorrhage, cerebral edema	Ceftriaxone (2 g/day), negative air pressure ventilation (7)	Death
Gear et al.^15^	Johannesburg	20/M	Complete blood count, urinalysis	Stung by an unknown agent	Malaise, chills, profuse sweating, frontal headache, nausea, vomiting, painful eyes, myalgia, hematochezia, Diffuse abdominal pain, malaise, erythematous maculopapular rash	Gastrointestinal hemorrhage, coagulopathy, Intrapulmonary hemorrhage, cardiorespiratory arrest, splenomegaly, enlarged axillary lymph nodes, hepatic necrosis, fulminating hepatitis, tubular necrosis in the kidney, fibrin deposition in glomeruli, necrosis in the spleen	IV ampicillin, chloroquine, chloramphenicol, fresh‐frozen plasma, platelets, blood transfusion, IV fluids, peritoneal dialysis (4)	Death
Gear et al.^15^	Johannesburg	19/F	Complete blood count, urinalysis	Direct human contact	Myalgia, diarrhea, vomiting, abdominal pain, rash	Tender and enlarged axillary lymph nodes	Heparin, Lassa fever antiserum, IV fluids (8)	Recovered
Gear et al.^15^	Johannesburg	20/F	Complete blood count, urinalysis	Direct human contact	Myalgia, diarrhea, abdominal pain, rash, vomiting	Tender and enlarged axillary lymph nodes, hemorrhage	Lassa fever antiserum, heparin, fresh‐frozen plasma, platelets, IV fluids (10)	Recovered
Kuming et al.^16^	Johannesburg	20/F	Fluorescent antibody test, tissue culture of aqueous	N/A	Lower back pain, high fever, injected conjunctivae	Hepatitis, mild DIC, uveitis (after 3 months)	Lassa fever antiserum (21)	Recovered
Timen et al.^17^	Netherlands	40/F	RT‐PCR	Exposure to bats	Chills, high fever	Liver failure, hemorrhage	Negative air pressure ventilation (5)	Death
Promed^18^	Colorado	44/F	Anti MARV IgM and IgG ELISA	Exposure to bats	severe headache, chills, nausea, vomiting, diarrhea	Hepatitis, renal failure, pancytopenia, coagulopathy, myositis, pancreatitis, encephalopathy	Ciprofloxacin, antiemetics, IV fluids, doxycycline (16)	Recovered
Ristanović et al.^19^	Yugoslavia	45/M	Histochemistry, electron microscopy	Accidental laboratory infection	Fever, chills, conjunctivitis, headache, dry cough, insomnia, nausea, vomiting, pruritus, dysphagia, odynophagia, pharyngeal spasm, oropharyngeal erythema, caked mucus, watery diarrhea, anorexia, rash on the upper chest, erythema of face, malaise, hyperacusis, scleral icterus, amplified deep tendon reflexes, kernig's sign, tongue fasciculations, nystagmus, Babinski sign	Myocarditis, pericardial effusion, leucocytosis, bilateral hand tremors, elevated blood urea, hyperchloremia, metabolic acidosis, electrolyte abnormalities, granular casts in urine, proteinuria, coagulopathy	Convalescent‐phase plasma, gamma globulin, antibiotics, IV fluids, nutritional support, analgesics, anxiolytics (25)	Recovered
Ristanović et al.^19^	Yugoslavia	44/F	Histochemistry, electron microscopy	Contact with body fluid	Chills, pain in her calves, mild fever, asthenia, headache, myalgia, cough, pharyngitis, loin pain, diarrhea, conjunctivitis, dehydration, edema, generalized erythema, vomiting, palatal enanthema	Uterine hemorrhage, leukopenia, hemoconcentration, metabolic acidosis, hypocalcemia, hypovitaminosis, elevated transaminase, transient secondary amenorrhea, secondary hypomenorrhea	Convalescent phase plasma (22)	Recovered
Ristanović et al.^19^	Yugoslavia	25/F	N/A	Contact with body fluids	Fever, headache, abdominal pain, nausea, malaise	Spontaneous abortion, vaginal hemorrhage	N/A	Death
Ristanović et al.^19^	Yugoslavia	40/F	ELISA	Direct human contact	High grade fever, hematemesis, diarrhea, headache, abdominal pain, myalgia, arthralgia, dysphagia, dyspnoea, conjunctivitis	N/A	N/A	Death
Ristanović et al.^19^	Yugoslavia	22/F	RT‐PCR	N/A	Purpura	N/A	N/A	Recovered
Ristanović et al.^19^	Yugoslavia	8 months/F	RT‐PCR	N/A	Fever, diarrhea, vomiting	Splenomegaly	N/A	Recovered
Nyakarahuka et al.^20^	Uganda	30/M	RT‐PCR, antigen detection, anti‐MARV IgM ELISA	N/A	Fever, vomiting, diarrhea, dysphagia, dyspnoea	Gastrointestinal hemorrhage	Ceftriaxone, artesunate (6)	Death
van Paassen et al.^21^	Netherlands	41/M	RT‐PCR	Exposure to bats	Headache, myalgia, rash, relative bradycardia, hearing loss	Liver failure, systemic inflammatory response syndrome, shock, cerebral edema	Antibiotics, IV fluids, mechanical ventilation (9)	Death
Nyakarahuka et al.^22^	Uganda	35/M	Nil	Exposure to bats	High grade fever, vomiting, diarrhea, malaise, abdominal pain, myalgia, headache, joint pain, hiccups, hematemesis, convulsions, loss of consciousness	N/A	N/A	Death
Nyakarahuka et al.^22^	Uganda	50/F	RT‐PCR	Direct human contact	Fever	Hemorrhage	N/A	Death
Luke Nyakarahuka. et al.^22^	Uganda	37/M	RT‐PCR	Direct human contact	Fever, malaise, abdominal pain, lack of appetite, joint pains, hematemesis	N/A	N/A	Death
Luke Nyakarahuka. et al.^22^	Uganda	31/F	Anti‐MARV IgM, IgG ELISA	Direct human contact	Miscarriage, headache, vomiting, malaise, abdominal pain	Hemorrhage	N/A	Recovered

Abbreviations: DIC, disseminated intravascular coagulation; ELISA, enzyme‐linked immunosorbent assay; IV, intravenous; MARV, Marburg virus; PT, prothrombin time; PTT, partial thromboplastin time; RT‐PCR, reverse transcriptase polymerized chain reaction.

### 2.5 Quality assessment

Joanna Briggs Institute Critical Appraisal tool (JBI) for case reports and case series was implemented to critically appraise the included studies. The Risk of bias was assessed by eight authors independently. The Risk of bias of studies was reported based on the following cut‐off: low risk of bias if 70% of answers scored yes, a moderate risk if 50%–69% of questions scored yes, and a high risk of bias if yes scores were below 50%. Six studies reported a low risk of bias, and five studies reported a moderate risk of bias.

### 2.6 Statistical analysis

All data were extracted onto a predesigned Excel sheet and represented in percentages, mean, and standard deviation for appropriate variables.

## 3 RESULTS

**Figure 1:**
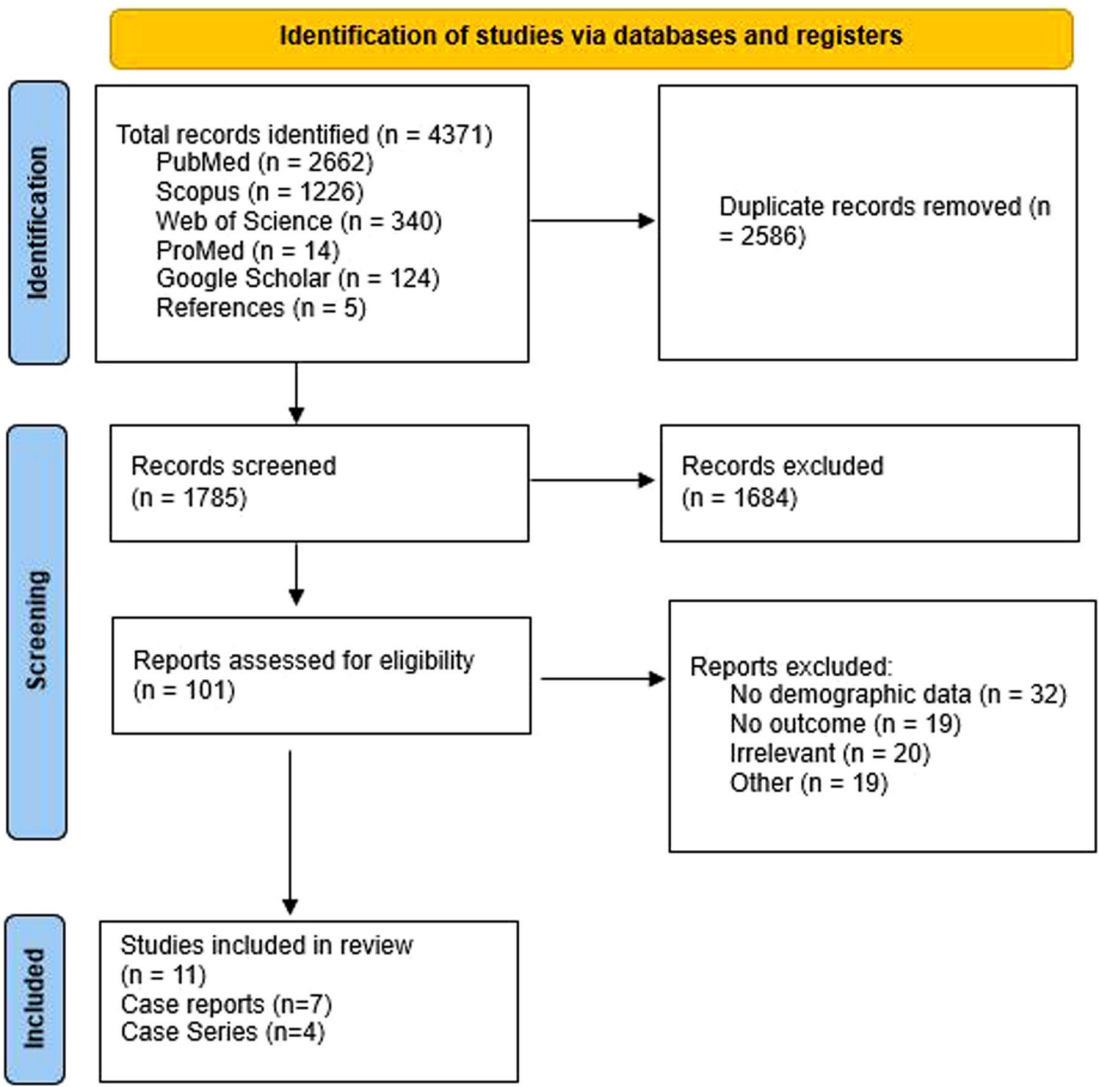
Search results from different databases.

A total of 11 studies (7 case reports and 4 case series) were included in the final analysis, and 21 cases of MVD were analyzed. Data from the included studies are presented in Table 1. The selection process of articles is shown in the PRISMA diagram (Figure 1).

### 3.1 Patient characters

Men constituted 7 (33.33%) out of the total 21 cases; the mean age was 32.22 years (SD = 13.88). Out of 21 cases, 2 (9.52%) cases were from Kenya, 5 (25%) cases were from Uganda, 3 (14.28%) cases were from the Netherlands, 4 (19.04%) cases were from Johannesburg, 6 (28.57%) cases were from Yugoslavia, and 1 case was from Colorado.

### 3.2 Clinical presentation

Within 21 cases who were diagnosed with MVD the most common symptoms were fever (*n* = 14, 66.67%), vomiting (*n* = 12, 57.14%), headache (*n* = 11, 52.38%), diarrhea (n = 11, 52.38%), and pain, which includes abdominal pain, arthralgia, retro‐orbital pain, loin pain, and so forth (*n* = 10, 47.62%). In addition to that the occasional symptoms were malaise (*n* = 9, 42.85%), myalgia (*n* = 8, 38.09%), rash (*n* = 6, 28.57%), chills (*n* = 6, 28.57%), conjunctivitis (*n* = 5, 23.81%), nausea (*n* = 4, 19.04%), hematemesis (*n* = 4, 19.04%), dysphagia (*n* = 3, 14.28%). Other symptoms were anorexia (*n* = 2, 9.52%), jaundice (*n* = 2, 9.52%), dyspnea (*n* = 2, 9.52%), cough (*n* = 2, 9.52%), erythema (*n* = 2, 9.52%), hematochezia (*n* = 2, 9.52%).

### 3.3 Investigations

Out of the 21 cases reported, investigations were conducted in 19 cases (90.48%). The most commonly used diagnostic test was RT‐PCR, which detected the Marburg virus in eight patients (42.11%). ELISA was employed in four cases (21.05%) to detect antibodies. Additionally, electron microscopy, histochemistry, a complete blood count, and urinalysis were each performed in three patients (15.79%). Fluorescent antibody detection, tissue culture, and antigen detection were also done in a few cases.

### 3.4 Route of transmission

In 16 of the reported 21 patients, transmission routes were successfully determined. It is unclear how the remaining five patients were infected. Contact transmission was the most frequent mode of MARV infection (*n* = 8, 50%) of which, direct contact constituted six cases and bodily fluids account for the rest. Bats were the subsequent source of transmission, accounting for six cases (37.5%). There has been one instance of unintentional laboratory infection caused by primates' blood, and one from an unidentified sting.

### 3.5 Treatment

Out of the 21 cases reported, 13 cases received treatment. Antibiotics (ampicillin, chloroquine, chloramphenicol, doxycycline, ceftriaxone) (*n* = 8, 61.5%) were the first line of treatment. IV Fluids (*n* = 6, 46.1%), and transfusion of blood products (fresh frozen plasma, platelets) (*n* = 5, 38.4%) were common treatment modalities. Other infrequently administered treatments were heparin (*n* = 3, 23%), Lassa fever antiserum (*n* = 3, 23%), negative air ventilation (mechanical ventilation) (*n* = 3, 23%), convalescent‐phase plasma (*n* = 2, 15.3%). Artesunate, anxiolytics, analgesics, antiemetics, gamma globulin, peritoneal dialysis, and nutritional support were rarely used.

### 3.6 Complications

Among the 21 identified cases, complications were reported in 17 cases (80.9%). The most common complication was hemorrhage (*n* = 9, 60%), followed by coagulopathies (*n* = 5, 33.3%). Other complications were enlarged axillary lymph nodes (*n* = 3, 20%), liver failure (*n* = 3, 20%), hepatitis (*n* = 4, 20%), cerebral edema (*n* = 2, 13%), splenomegaly (*n* = 2, 13%), hepatic necrosis (*n* = 2, 13%), metabolic acidosis (*n* = 2, 13%), leukocytosis (*n* = 2, 13%), and renal failure (*n* = 2, 13%).

### 3.7 Outcome

Duration of treatment was reported in 15 out of 21 cases. The duration of treatment ranged from 1 to 25 days, with a mean of 10 days. Twelve deaths were reported out of 21 cases and the mortality rate was 57.1%.

## 4 DISCUSSION

### 4.1 Epidemiology of MARV

**Figure 2:**
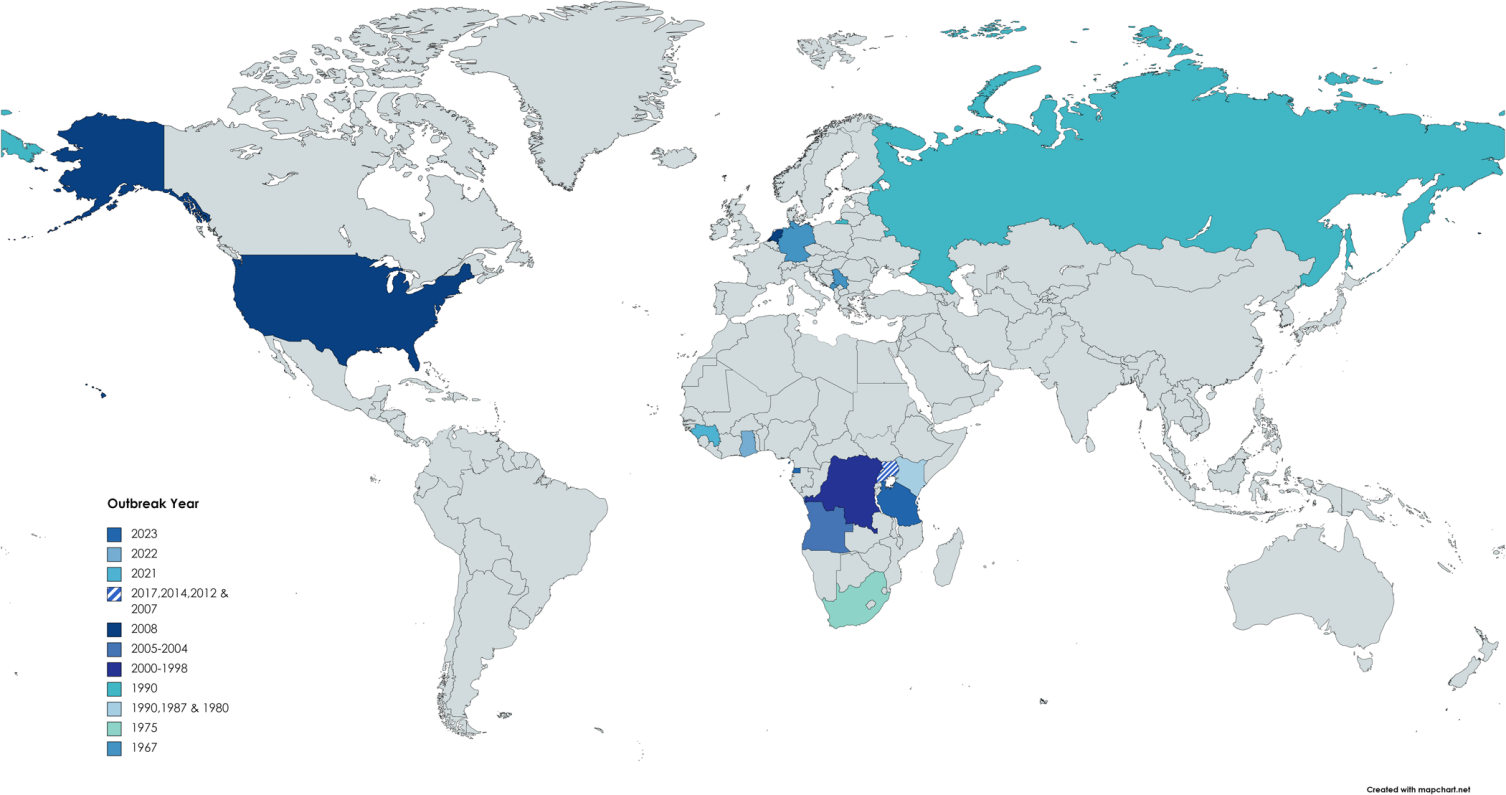
Map depicting the geographical distribution of MVD in various outbreaks. MVD, Marburg virus disease.

The most recent MARV outbreak, with 20 suspected cases and 9 confirmed cases, occurred in Equatorial Guinea in 2023. At the same time in March 2023, Tanzania also confirmed a single case of the MARV with eight probable cases. The epidemiological study of the outbreak is still in progress. The previous MVD outbreaks were in Guinea (2021) and Ghana (2022). Uganda experienced multiple outbreaks in 2017, 2014, 2012, and 2007. A single case of MARVs was reported in the Netherlands and the United States in 2008, which was believed to be occurred due to the spread of the infection from travelers of Uganda. Angola experienced the worst MARV outbreak ever, which was recorded in 2004–2005, with over 227 fatalities. Between 1998 and 2000, the Democratic Republic of the Congo also reported a sizable MARV outbreak. In Russia (1990), Kenya (1987 and 1980), and South Africa (1975), there were numerous minor outbreaks. Germany and Yugoslavia saw the MARV's first known outbreak in 1967. Figure 2 depicts countries affected by different outbreaks.

### 4.2 Pathophysiology

MARV enters the host cells via endocytosis, and the viral RNA genome is released into the host cytoplasm. The genome encodes seven structural proteins that are involved in viral replication, assembly, and immune evasion.^23^ Macrophages, monocytes, and dendritic cells are primarily affected by MARV. The virus enters hepatocytes through asialoglycoprotein; therefore, liver and lymphoid tissue are the principal targets of MARV. The virus also enters the cells through the folate receptor and DC‐SIGN (dendritic cell‐specific ICAM‐grabbing nonintegrin) receptors. Additionally, it leads to the necrosis of the follicles, medulla of the lymph node, and red pulp of the spleen, as well as the depletion of lymphocytes in the lymphoid tissue. MARV does not infect lymphocytes (T, B, and Natural killer cells). Monocytes and other mononuclear phagocytic cells have been shown to activate infection leading to a cytokine storm. Studies have shown that MARV‐infected animals exhibit greater levels of Interferon and Interleukin‐6, which results in increased vascular permeability and defective coagulation are the results of these factors together.^24^

TNF‐enhanced (tumor necrosis factor) endothelial cell permeability, the depletion of fibrinogen, and lower levels of factors V and II are the causes of thrombocytopenia seen in Marburg hemorrhagic fever (MHF). In addition to coagulation abnormalities like disseminated intravascular coagulation, and systemic viral dissemination, it also causes multiorgan failure in severe MHF patients.

Further, the innate immune system is paralyzed by MARV's blockage of the interferon pathway, which is similar to the evasion of the immune system in the Ebola virus disease.^24^

### 4.3 Diagnostic modalities

The diagnosis of the MARV can be made by various laboratory diagnostic methods. In the early stages of the illness, detection of the virus antigen is appropriate, as high titers of the viral antigens are present in the blood. This can be done via virus isolation by cell culture, antigen capture enzyme‐linked immunosorbent assay (ELISA), or immunohistochemical analysis. For antigen capture ELISAs, the antibodies used are monoclonal antibodies to the recombinant nucleoprotein of Lake Victoria MARV, specifically, MAb2A7 and MAb2H6. In relatively later stages, diagnosis can be made by serology via the detection of IgM and IgG antibodies. This can be done by indirect immunofluorescence or IgM and IgG capture ELISA. Molecular diagnostic methods, such as reverse transcription polymerase chain reaction (RT‐PCR), nested RT‐PCR, and real‐time quantitative RT‐PCR, have been found to be sensitive and specific for the diagnosis of MARV and can be used to diagnose MARV in the early and late stages of the disease. Thrombocytopenia and leukopenia are seen in complete blood picture of MVD. Increase in the liver enzymes, aspartate aminotransferase (AST), and alanine aminotransferase (ALT) are characteristic, with associated coagulation defects. On urinalysis, proteinuria is often detected due to renal dysfunction.^25^, ^26^, ^27^

### 4.4 Routes of transmission

The primary mode of transmission of MVD is zoonotic transmission. Rousettus aegyptiacus and *Hipposideros caffer* are the important species of bats associated with the transmission of the virus.^1^ Direct contact with secretions, exposure to aerosols, and consumption of bushmeat are potential sources of infections. Direct transmission from infected patients with their secretions or excretions is also a possibility. Healthcare workers and relatives of the infected can be infected through aerosol or direct contact with body fluids, sweat, blood, and so forth.^28^, ^29^ Transmission of the virus by laboratory accidents due to negligence has been reported in a few cases.

**Figure 3:**
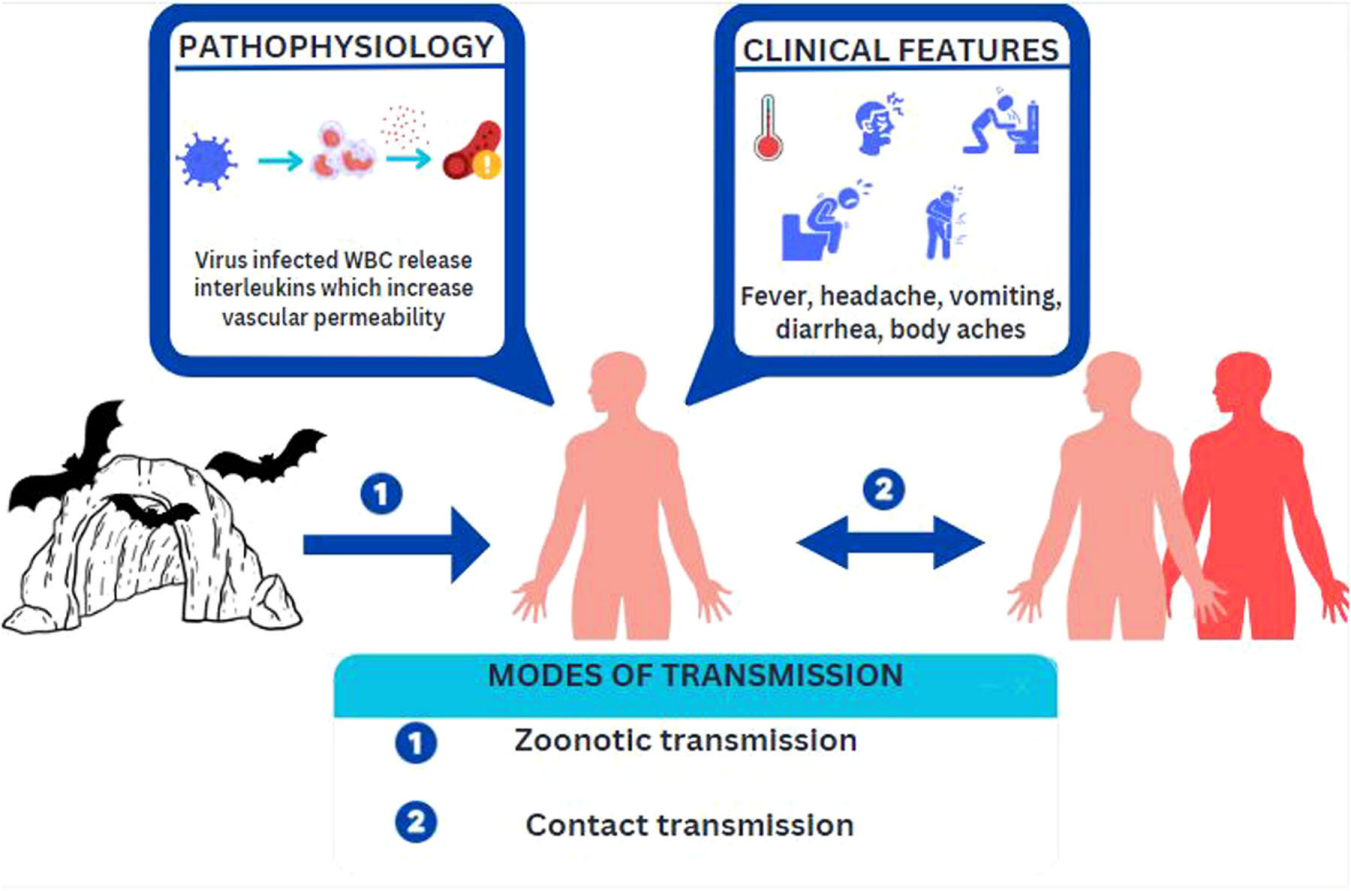
Pathophysiology, clinical features, and modes of transmission of the Marburg virus disease.

Currently, there are no vaccines or antiviral treatments approved for MVD. However, supportive care, including rehydration with oral or intravenous fluids and treatment of specific symptoms, improves survival. Figure 3 summarizes the pathophysiology, clinical presentation, and modes of transmission of the disease.

### 4.5 Recommendations

#### 4.5.1 For natives and travelers

While visiting mines or caves inhabited by fruit bats (*Rousettus aegyptiacus*), wearing protective gear and avoiding contact with bats is recommended. During outbreaks, inhabitants and travelers must avoid close physical contact and contact with infected blood and bodily fluids, including sweat, blood, urine, feces, vomit, breast milk, amniotic fluid, vaginal secretions, and semen, as well as any objects that may have come into contact with the infected. Using personal protective equipment, good hygiene while nursing or visiting the sick, and safe funeral practices will help prevent this. Contacts must monitor themselves for 21 days, quarantine themselves, and immediately seek medical attention if required.^30^, ^31^

#### 4.5.2 For healthcare professionals

Clinicians should be aware of the potential for imported cases. It is important to systematically assess patients for the possibility of viral hemorrhagic fevers through a triage and evaluation process, including a detailed travel history. The patient must be isolated at a healthcare facility in a single room with a private bathroom/covered bedside commode). Trained and equipped healthcare workers are only to be used and a register with the names of people entering the patient's room must be maintained. Healthcare workers must adhere to infection prevention and control procedures to prevent transmission, including wearing appropriate personal protective equipment (PPE). Healthcare personnel can be infected through contact with patient's body fluids, contaminated medical supplies, and equipment, or contaminated environmental surfaces. Splashes to unprotected mucous membranes (e.g., the eyes, nose, or mouth) are particularly hazardous. Procedures that can increase environmental contamination with infectious material, which include handling potentially contaminated needles or other sharps or aerosol generation should be minimized. Only necessary tests and procedures must be performed. Cases must be notified to the facility's infection prevention control program.^32^

## 5 LIMITATIONS

The results of our systematic review and the accuracy of our findings are seriously impeded by the paucity of literature on MVD. Several case reports and case series were excluded due to a lack of demographic data and incomplete information. Treatment was not reported in most of the case reports which increases the risk of biased information. The absence of cohort and case–control studies on MVD is a major limitation of the study. Our study provides a current, exploratory overview of the data even though the data from more extensive, well‐designed studies are not yet accessible.

## 6 CONCLUSION

The current situation in the United Republic of Tanzania and Equatorial Guinea with the declaration of an outbreak is alarming. The MARV causes severe illness and is associated with high case fatality rates. The most commonly observed route of transmission is facilitated through direct physical contact, which can occur via bodily fluids, sexual activity, droplets from infected individuals, and contaminated fomites. The most common symptoms were fever, vomiting, headache, diarrhea, and pain, and the frequently used investigation methods were RT‐PCR and ELISA. Antibiotics are the first line of treatment, but currently, there are no vaccines or antiviral treatment modalities available for MVD. It is crucial to have a comprehensive understanding of its epidemiology, clinical manifestations, and effective diagnostic and treatment measures for MVD to prevent the risk of widespread transmission on a global scale. This systematic review introduces a novel approach by compiling various parameters to have a well‐rounded approach.

## AUTHOR CONTRIBUTIONS

**Deekshitha Alla**: Conceptualization; data curation; formal analysis; investigation; methodology; project administration; resources; software; supervision; writing—review & editing. **Sai Sri Hari Paruchuri**: Data curation; methodology; software; writing—original draft. **Angad Tiwari**: Data curation; methodology; writing—original draft. **Sai Santhosha Mrudula Alla**: Conceptualization; formal analysis; methodology; software; supervision; writing—review & editing. **Rakesh Thulaseedharan Pillai**: Data curation; methodology; writing—original draft. **Bandakadi Sandeep Kumar Reddy**: Data curation; methodology; writing—original draft. **Anju Pradeep**: Data curation; software; writing—original draft. **Dhruv Jayeshkumar Shah**: Data curation; software; writing—original draft. **Mert Sabıroğlu**: Data curation; writing—original draft. **Sachi chavda**: Data curation; writing—original draft. **Patrick Biziyaremye**: Conceptualization; writing—review & editing.

## CONFLICT OF INTEREST STATEMENT

The authors declare no conflict of interest.

## TRANSPARENCY STATEMENT

The lead author Patrick Biziyaremye affirms that this manuscript is an honest, accurate, and transparent account of the study being reported; that no important aspects of the study have been omitted; and that any discrepancies from the study as planned (and, if relevant, registered) have been explained.

## Data Availability

The data that support the findings of this study are available from the corresponding author upon reasonable request.
